# Building a culture of connection in early childhood education: The Hand in Hand Foundations Course

**DOI:** 10.1002/imhj.70030

**Published:** 2025-06-23

**Authors:** Angela Sillars, Ahava Vogelstein, Pamela J. Oatis, Annie Davis Schoch, Anna Cole, Pamala Trivedi, Maya Coleman

**Affiliations:** ^1^ Vermont State University Castleton Vermont USA; ^2^ Hand in Hand Parenting San Francisco California USA; ^3^ FAAP, Mercy St. Vincent Medical Center, Nationwide Children's Hospital Toledo Toledo Ohio USA; ^4^ Child Trends Bethesda Maryland USA; ^5^ Hand in Hand Parenting, University of Portsmouth Portsmouth Hampshire UK; ^6^ Department of Pediatrics Georgetown University Washington USA; ^7^ Department of Pediatrics Hand in Hand Parenting, Georgetown University Washington USA

**Keywords:** connection, early childhood education, reflective practice, socioemotional learning, teacher well‐being, teacher‐child relationships, التعليم في مرحلة الطفولة المبكرة، رفاهية المعلم، العلاقات بين المعلم والطفل، التعلم الاجتماعي‐العاطفي، الممارسة التأملية, 早期儿童教育, 教师福祉, 师生关系, 社会情感学习, 反思实践, Éducation de la petite enfance, bien‐être des enseignants, relations entre les enseignants et les enfants, apprentissage socio‐émotionnel, pratique réfléchie, Frühkindliche Bildung, Wohlbefinden von Erziehern, Erzieher‐Kind‐Beziehung, sozioemotionales Lernen, Reflektion, 幼児教育、教師のウェルビーイング、教師と子どもの関係、社会的情動的学習、内省的実践, educación en la temprana niñez, bienestar del maestro, relaciones entre el maestro y el niño, aprendizaje socioemocional, práctica con reflexión

## Abstract

Healthy early childhood development unfolds in the context of relationships with important caregivers, including early educators. Grounded in the evidence on early relational health, the 8‐week Hand in Hand Foundations Course for Early Childhood Educators teaches a novel connection‐based approach to understanding and responding to young children's emotions. Educators learn five tools for bolstering connection during emotional experiences (Staylistening), increasing positive and playful interactions with children (Special Time, Playlistening), responding to challenging behavior (Setting Limits), and creating sustainable, peer‐supported reflective practice groups (Listening Partnerships). The goal is to foster stronger educator‐child relationships, improve well‐being for educators and children, and build a culture of connection and reflection in ECE. This mixed‐methods pilot study of the Foundations Course in the United States investigated educators’ experiences with the course, soliciting input on the feasibility of using the tools in the classroom and areas for improving the course. Educators reported that the theories resonated with them, they incorporated many of the tools, and they saw benefits for children and the ECE community. They also expressed a need for ongoing learning opportunities, implementation scaffolding, and organizational support. These findings have implications for supporting educator and child social‐emotional health and potential future revisions for the course.

## خلاصة

بناء ثقافة التواصل في التعليم في مرحلة الطفولة المبكرة: دورة الأساسيات من مؤسسة “هاند إن هاند” الملخص يتطور النمو الصحي للأطفال في مرحلة الطفولة المبكرة ضمن سياق العلاقات مع مقدمي الرعاية الأساسيين، ومن بينهم المعلمون الأوائل. واستنادًا إلى الأدلة العلمية حول الصحة العلائقية المبكرة، تقدم دورة “الأساسيات” من مؤسسة (هاند إن هاند) ، وهي برنامج تدريبي مدته 8 أسابيع لمعلمي الطفولة المبكرة، نهجًا جديدًا قائمًا على التواصل لفهم مشاعر الأطفال الصغار والاستجابة لها. يتعلم المعلمون خمس أدوات تعزز التواصل أثناء التجارب العاطفية (الاستماع الداعم)، وتزيد من التفاعل الإيجابي والمرح مع الأطفال (الوقت الخاص، الاستماع من خلال اللعب)، وتساعد في التعامل مع السلوكيات الصعبة (وضع الحدود)، إضافة إلى إنشاء مجموعات مستدامة من الممارسات التأملية المدعومة من الزملاء (شراكات الاستماع).يهدف البرنامج إلى تقوية علاقات المعلم‐الطفل، وتحسين رفاهية المعلمين والأطفال، وبناء ثقافة من التواصل والتفكر داخل بيئة التعليم في مرحلة الطفولة المبكرة.تبحث هذه الدراسة التجريبية ذات المنهج المختلط في الولايات المتحدة تجارب المعلمين مع الدورة، كما تستعرض آراءهم حول مدى إمكانية تطبيق الأدوات داخل الصفوف، والمجالات المحتملة لتطوير البرنامج.أفاد المعلمون بأن المبادئ النظرية كانت مؤثرة بالنسبة لهم، وأنهم استخدموا العديد من الأدوات، ولاحظوا فوائد ملموسة للأطفال ولمجتمع الطفولة المبكرة. كما أشاروا إلى حاجتهم إلى فرص تعلم مستمرة، وأدوات داعمة للتنفيذ، ودعم تنظيمي. وتُسلّط هذه النتائج الضوء على أهمية دعم الصحة الاجتماعية والعاطفية لكل من المعلم والطفل، مع إمكانية إجراء تعديلات مستقبلية على الدورة.

## 摘 要

健康的早期儿童发育是在与重要看护者 (包括学前教育工作者) 的关系中逐步展开的。基于“早期关系健康”的实证研究, “手拉手学前教育工作者基础课程”为期八周, 教授一种以联结为核心的新方法, 用于理解并回应幼儿的情绪。教育工作者学习五种策略工具, 包括在儿童情绪体验中增强联结的“倾听陪伴”、增加与儿童积极游戏互动的“专属时间”和“游戏倾听”、应对挑战行为的“设定界限”, 以及建立可持续的、由同伴支持的反思实践小组“倾听伙伴关系”。课程目标在于促进教育工作者与幼儿之间更紧密的关系, 提升教育工作者和儿童的整体福祉, 并在学前教育领域建立一种联结与反思的文化。本研究是一项在美国开展的混合方法试点研究, 旨在调查教育工作者对该基础课程的体验, 并征求他们关于在课堂中使用这些工具的可行性以及课程改进方向的意见。教育工作者普遍认为这些理论引起了他们的共鸣, 他们采用了其中的许多工具, 并观察到这些工具对儿童和学前教育群体均带来了积极影响。同时, 他们也表达了对持续的学习机会、实施支持机制以及组织层面支持的需求。研究结果对于促进教育工作者与儿童的社会情感健康以及该课程的未来优化具有重要意义。

## Abstraite

Le développement sain dans la petite enfance s'effectue dans le contexte des relations avec les personnes importantes qui s'occupent d'enfants, y compris les éducateurs préscolaires. Fondé sur les données probantes de la santé relationnelle précoce, la formation de 8 semaines Hand in Hand Foundations pour éducateurs de la petite enfance enseigne une approche novatrice fondée sur les liens pour comprendre et répondre aux émotions des jeunes enfants. Les éducateurs développent cinq outils pour renforcer la connexion au cours d'expériences émotionnelles (Staylistening), pour accroître les interactions positives et ludiques avec les enfants (Special Time, Playlistening), pour réagir à des comportements difficiles (Setting Limits) et pour créer des groupes de pratique de réflexion, durables et basés sur une réflexion de groupe (partenariats d’écoute). Le but est d'encourager des liens plus solides entre l’éducateur et l'enfant, d'améliorer le bien‐être des éducateurs et des enfants, et de créer une culture de connexion et de réflexion dans le domaine de l’éducation des très jeunes enfants. Cette étude pilote à méthodes mixtes de cette Formation aux principes de Fondations, aux États‐Unis, a examiné l'expérience des éducateurs avec cette formation, en sollicitant leurs commentaires sur la possibilité de l'utilisation de ces outils dans la salle de classe et ainsi que sur les domaines pouvant améliorer la formation. Les éducateurs ont indiqué que les théories les touchaient, qu'ils incorporaient bon nombre de ces outils et qu'ils en voyaient les avantages pour les enfants et l'enseignement aux jeunes enfants. Ils ont également exprimé leurs espoirs de pouvoir participer à des formations continues, de pouvoir mettre en œuvre un échafaudage de mise en œuvre et de pouvoir recevoir un soutien organisationnel. Ces résultats ont des implications pour le soutien de la santé socio‐émotionnelle de l’éducateur et de l'enfant, ainsi que pour les révisions futures possibles de cette formation.

## Abstrakt

Eine gesunde frühkindliche Entwicklung entfaltet sich im Kontext von Beziehungen zu wichtigen Bezugspersonen, zu denen auch Erzieherinnen und Erzieher gehören. Der 8‐wöchige Hand‐in‐Hand‐Grundlagenkurs für Erzieherinnen und Erzieher vermittelt einen neuartigen, bindungsbasierten Ansatz, um die Emotionen von Kleinkindern zu verstehen und auf sie zu reagieren. Erzieherinnen und Erzieher lernen fünf Tools, um die Bindung während emotionaler Erlebnisse zu stärken (Staylistening), positive und spielerische Interaktionen mit Kindern zu fördern (Special Time, Playlistening), auf herausforderndes Verhalten zu reagieren (Setting Limits) und nachhaltige, von Gleichaltrigen unterstützte Reflexionsgruppen zu bilden (Listening Partnerships). Ziel ist es, stärkere Erzieher:innen‐Kind‐Beziehungen zu fördern, das Wohlbefinden von Erzieher:innen und Kindern zu verbessern und eine Kultur der Verbundenheit und Reflexion in der Kinderbetreuung aufzubauen. Diese mixed‐methods‐Pilotstudie zum Grundlagenkurs in den USA untersuchte die Erfahrungen der Erzieherinnen und Erzieher mit dem Kurs und bat um Beiträge zur Durchführbarkeit des Einsatzes der Tools im Klassenzimmer und zu Verbesserungsmöglichkeiten des Kurses. Die Pädagog:innen berichteten, dass die Theorien bei ihnen auf Resonanz stießen, dass sie viele der Tools einsetzten und dass sie einen Nutzen für die Kinder und das Feld frühkindlicher Bildung und Betreuung sahen. Sie äußerten auch den Bedarf an fortlaufenden Lernmöglichkeiten, an Unterstützung bei der Umsetzung und an organisatorischer Unterstützung. Diese Ergebnisse haben Implikationen für die Förderung der sozial‐emotionalen Gesundheit von Erzieher:innen und Kindern sowie für mögliche zukünftige Überarbeitungen des Kurses.

## 抽象的な

乳幼児期の健全な発達は、幼児教育者を含め、重要な存在である養育者との関係の中で展開される。幼児教育者のための8週間のHand in Hand基礎コースは、乳幼児期の対人関係の健全性に関するエビデンスに基づく、幼い子どもたちの情動についての理解と応答をめざす、つながりを基盤とした斬新なアプローチを学ぶ。教育者は、情動的な体験の中でつながりを強化する (Stay listening) 、子どもとの前向きで遊び心のある交流を増やす (Special Time、Play listening) 、困難な行動に対応する (Setting Limits) 、持続可能で仲間同士が支え合う内省的実践グループを作る (Listening Partnerships) という、 5 つのツールを学ぶ。このコースの目的は、教育者と子どもとの関係性をより強固にし、教育者と子どもの幸福感を向上させ、ECE(Early Childhood Education 幼児教育)におけるつながりと内省の文化を構築することである。米国における基礎コースのこの混合方法によるパイロット研究では、教育者のコースでの経験を調査し、教室でツールを使用することの実現可能性とコースを改善するための領域について検証した。教育者たちは、理論に共鳴し、ツールの多くを取り入れ、子どもたちやECEコミュニティにとって有益であると報告した。彼らはまた、継続的な学習の機会、実践の足場、組織的な支援の必要性を表明した。これらの知見は, 教育者と子どもの社会性と情動の健康を支援するための手がかりとなり、今後のコース改訂に影響を与える可能性がある。

## Resumen

El desarrollo de una temprana niñez saludable se desenvuelve en el contexto de relaciones con cuidadores importantes, incluyendo los primeros educadores. Con base en la evidencia sobre la salud de la temprana relación, el Curso de Fundaciones Mano a Mano para Educadores de la Temprana Niñez, de 8 semanas, enseña un acercamiento novedoso con base en la conexión para comprender y responder a las emociones de los niños pequeños. Los educadores aprenden cinco herramientas para reforzar la conexión durante las experiencias emocionales (Mantenerse Escuchando), aumentar las interacciones positivas y juguetonas con los niños (Tiempo Especial, Jugar Escuchando), responder a comportamientos desafiantes (Establecer límites), y crear grupos sostenibles de práctica con reflexión y apoyo de compañeros (Asociarse para Escuchar). El objetivo es fomentar relaciones más fuertes entre educador y niño, mejorar el bienestar de educadores y niños, así como crear una cultura de conexión y reflexión en la Educación en la Temprana Niñez (ECE). Este estudio piloto con métodos combinados del Curso de Fundaciones en los Estados Unidos investigó las experiencias de los educadores con el curso, solicitando puntos informativos sobre la posibilidad de usar las herramientas en la sala de clases y áreas para mejorar el curso. Los educadores reportaron que las teorías resonaban dentro de ellos, que ellos incorporaron muchas de las herramientas y que vieron beneficios para los niños y la comunidad ECE. Ellos también expresaron la necesidad de oportunidades de un aprendizaje continuo, implementar un sistema que les enseñe a los niños a hacer sus tareas independientemente, así como el apoyo de organizaciones. Estos resultados tienen implicaciones para apoyar la salud socioemocional del educador y del niño y revisiones futuras potenciales para el curso.

## 1 INTRODUCTION

Early childhood development is a stage of unparalleled importance that lays the foundation for children's future learning, wellbeing, and connection with others (Center on the Developing Child, 2016; Shonkoff, 2010). Early development unfolds within the context of a young child's relationships with the adults who care for them (Sameroff, 2009). In the presence of positive, reciprocal, and responsive interactions between caregivers and young children, both parties experience improved emotional well‐being, and young children form a strong foundation of early relational health (Willis & Eddy, 2022). Concerted attention to preserve and bolster the innate, but all‐too‐often subverted, human drive for social connection is vital from earliest development to combat what Surgeon General Vivek Murthy termed an “epidemic of loneliness and isolation” that is disproportionately impacting children and youth (Office of the Surgeon General [OSG], 2023). For many young children, in addition to parents/caregivers, some of their most important early connections are with early educators. The building blocks of these relationships are sensitive, responsive interactions, wherein educators meet children's needs for closeness, safety, and warmth—especially during moments of heightened emotions (Blewitt et al., 2020). Bolstered by the science and principles of early relational health, federal guidance suggests investing in programs that support the mental health of young children and their educators via relationship‐based interventions (Administration for Children & Families, 2024). The current study reports the pilot study results for the Hand in Hand (HiH) Foundations Course for Early Childhood Educators, which was designed to reimagine connection and emotional expression in the ECE classroom.

### 1.1 The emotional context of the classroom for children

In early childhood, it is developmentally appropriate for emotions to be rapidly changing and strong. The classroom environment and teacher‐child interactions are replete with a range of emotions. As their brains develop the fundamental architecture of the stress response, children need support from adults to navigate emotional moments (Shonkoff, 2010). Educators engage in many emotion‐laden tasks with children daily, each of which provides opportunities for connection. They are therefore key socializers of children's emotional development, communicating directly and indirectly about how, when, and to what extent children are encouraged to express emotions (Denham et al., 2012; Poulou & Denham, 2023). Teachers’ responses to children's emotions depend upon their own upbringing, culture, and beliefs about emotional expression (Catherine & Swadener, 2021). Adults can, for example, react in ways that accept and validate children's emotions, or that dismiss and disapprove of them (Gottman et al., 1996; Fatahi et al., 2023).

**Key Findings**

1. The Hand in Hand Foundations Course for Early Childhood Educators teaches a novel theory of the adaptive function of emotions and five tools that fundamentally shift educators’ role during children's emotional expression, focused on promoting attuned listening, authentic connection to children, sustainable peer support with other adults, and reflective practice.
2. In this pilot study, educators reported that they learned and integrated this new theory into their work. This was evident in their attuned listening to children and other adults, and the corresponding perceived impact on relationships and their own well‐being.
3. The educators made suggestions for helping future educators who participate in the Foundations Course to better integrate these “revolutionary” tools into their everyday practices, including training entire centers together to encourage community support and sustainability.

**Statement of relevance**

In the early childhood field, there is substantial research affirming the importance of strong teacher‐child relationships and educator well‐being. This article is the first to present evidence on the benefits of the Hand in Hand Foundations Course for Early Educators, which teaches a novel theory of children's emotions and five attuned listening tools. Educators report enhanced interactions, reflective capacity, and social support—all of which are needed now more than ever amid US society's “epidemic of loneliness.”

The ways that educators respond to children's emotions have implications for creating a trauma‐informed, healing centered, and liberatory classroom environment. Children who have experienced early adversity do best when they feel safe and secure in their relationship with their teacher, who can stay attuned and responsive when the child may be upset or stressed (Chudzik et al., 2023; Sciaraffa et al., 2018). However, other dynamics can interfere with the development of these kinds of relationships, particularly at the intersections of marginalized identities (e.g., class, race, language, gender and sexuality, citizenship). For example, in the context of our racialized social structures, implicit biases can lead to overly negative interpretations of Black children's emotions, which can lead to more negative responses such as punishment and exclusionary discipline (Halberstadt et al., 2022; Meek & Gilliam, 2016). Young children with disabilities also experience disproportionate rates of exclusionary discipline—especially those whose behavior is characterized by perceived emotional or behavioral dysregulation (Zeng et al., 2021). In contrast, an affirming classroom environment is characterized by acceptance of different types of emotional expression as well as regular self‐reflection by educators (Catherine & Swadener, 2021).

### 1.2 The emotional context of the classroom for educators

Clearly, classroom interactions pertaining to emotions are important. They also demand a considerable amount of reflective capacity and patience on the part of the educator. There is widespread empirical agreement that educators' well‐being is linked with the quality of their relationships with children and the warmth of their interactions (Smith & Lawrence, 2019). Specifically, educators can best provide emotionally supportive care when they are mindful, self‐reflective, emotionally well‐resourced, and when they feel satisfied in their jobs (Becker et al., 2017; Denham et al., 2017; Jennings, 2015). Unfortunately, these conditions are often not the reality for the early childhood education profession (Kwon et al., 2021). These educators often do not earn a living wage, may lack access to health care and other critical benefits, and experience depression and burnout at a higher rate than their peers in K‐12 settings (Lessard et al., 2020; McLean et al., 2021; Otten et al., 2019). While their work is rewarding, it is also emotionally and physically intense and often under‐resourced (Cumming, 2017; Farewell et al., 2022; Kwon et al., 2021). Workplace stressors predict less supportive interactions with children and more educator‐child conflict (Denham et al., 2017; Whitaker et al., 2015), which in turn worsen educator stress (Gagnon et al., 2019).

In response to this troubling pattern, a range of supports have been designed to assist educators in navigating the emotional terrain of their jobs, which includes their own emotional state, their relationships with children, and child behavioral challenges (Sabol & Pianta, 2012). Either directly, by providing skills for interacting with children, or indirectly, by improving teacher well‐being, such supports can promote sensitive interactions and positive relationships among educators and young children (Bierman et al., 2018; Blewitt et al., 2020).

### 1.3 The Hand in Hand approach

The Hand in Hand Foundations Course for Early Childhood Educators (referred to subsequently as “the Foundations Course”) is one such resource through which educators expand their capacity to interact with children in a sensitive and intentional manner. The 8‐week Foundations Course teaches early educators a theory of emotional functioning and five connection‐based caregiving tools. This approach, developed by an international nonprofit called Hand in Hand Parenting, was initially developed over 30 years ago as a support for parents in a cooperative preschool setting. The HiH approach is based on the premise that, to resolve societal issues of violence and injustice, people must have opportunities to connect, to have their needs met, and to address the lingering effects of adversity. The tools were therefore designed to bolster and protect people's innate instincts to connect and to heal.

The HiH approach consists of (1) a theory of interpersonal connection and (2) five “listening” tools that operationalize this theory. The HiH theory of connection highlights the power of attuned listening to facilitate emotional growth and promote adaptive, healing‐centered responses to challenging experiences and adversity. HiH conceptualizes emotional expression, often accompanied by external somatic indicators (e.g., talking, crying, laughing, shaking, yawning), as part of a metaphorical “emotional digestive system” in which humans offload tension and clear out the emotional residue of challenging experiences. Humans can process emotions through this innate and intuitive system when in connection with a caring, attuned listener. The role of the listener is, then, to accompany another person during their emotional experience, offering positive regard and warm connection as their attention serves as an anchoring element during these emotional moments. After these moments, individuals are able to access increased adaptability, flexible thinking, and creativity (Wipfler & Schore, 2016).

The five HiH tools are called “listening tools” because they provide structured ways to offer warm connection, responsiveness, and careful stewardship of the emotional experiences of others (see Table 1). Four of the tools (Special Time, Staylistening, Setting Limits, Playlistening) are used in interactions between adults and children, and the fifth tool (Listening Partnerships) is a self‐reflective, peer‐support tool for adults. In this way, the HiH approach intentionally mobilizes a “parallel process” whereby the adult receives emotional support from a mentor and responsive peers, which in turn grows their capacity to provide it to children These tools were developed based on the science of early relational health and neuroscience (Wipfler & Schore, 2016).

**TABLE 1:** The five Hand in Hand listening tools.

Tool	Description
Listening partnerships	Listening Partnerships are modeled in the mentoring calls and then implemented in a peer‐to‐peer model with colleagues after the course. A Listening Partnership is a structure in which two or more adults can take turns providing mutual emotional support to one another while they are engaged in a self‐reflective process that facilitates emotional experience and emotional expression. Adults take equal turns listening to each other without interrupting or offering advice. A timer is set so that each person receives equal time and to signify when to end. At the end, they use a playful “refresher question” to bring the participants back to the present moment to help them transition away from potentially distressing topics. This peer‐to‐peer experience produces high‐quality connection, social support, and emotional healing
Staylistening	Staylistening is an approach through which adults offer children support and connection during emotional moments. The adult offers attention, connection, and eye contact while listening to the child's feelings and emotional expression. During Staylistening, adults set and hold limits on off‐track behavior as needed. This tool facilitates children's emotional recovery from hard moments so that they can access their cooperation and problem‐solving skills again.
Setting Limits	The Setting Limits tool supports the adult to address off‐track behavior using boundary setting early and often while staying warmly connected to the child throughout the experience. Setting Limits is a 3‐step process: 1) Listen to understand the signal the child is sending and the needed limit, 2) set the limit, and 3) listen again as you hold the limit firmly with warmth. Setting Limits occurs within an episode of Staylistening.
Special time	Special Time is one‐on‐one, child‐led playtime. During Special Time the adult follows the child's lead in play with delight, while offering consistent high quality connection and avoiding judgment or scaffolding. Special Time allows the child to be in charge in a managed way where they can feel confident in the adult's attention, and for the adult to connect with the child through the language of play. Special Time is a protected time, labeled by the adult, and is timed. The goal is to build and maintain a strong, high quality connection with each individual child.
Playlistening	Playlistening is an approach to interactions in which an adult takes the less powerful role in play, often using silliness to facilitate laughter (without tickling) and to diffuse tension. The goal is to use laughter and playfulness to promote the emotional recovery process and specifically utilize laughter to begin the healing process.

The HiH approach is currently used internationally by more than 250 Certified Instructors on six continents in 15 languages as a tool to promote early relational health. In 2023, Hand in Hand delivered 135 courses for parents and early childhood professionals, and over 16,000 parents and professionals accessed Hand in Hand programs and/or services. While there have not been large‐scale, rigorous studies of the Hand in Hand parenting program, there have been several case studies affirming its value to the parents and communities served (Association for Maternal and Child Health Programs [AMCHP], 2022). The current study represents a significant step forward in creating an evidence base for HiH.

### 1.4 The Hand in Hand Foundation Course

While parents comprise a large portion of HiH's portfolio, the educator‐facing services and programs are growing rapidly (Hand in Hand Parenting, 2023). The Foundations Course is an 8‐week course that integrates didactic and experiential components to share the HiH theory and listening tools in a manner that is specifically adapted for early educators. The Foundations Course integrates didactic content (i.e., video and written materials) and a weekly cohort‐based mentoring video call. During the mentoring calls, instructors provide guidance and support as educators share their experiences applying the five tools in their setting, and educators provide peer‐to‐peer emotional support through the listening tool (see Appendix A for course syllabus). Concurrent with the mentoring calls, instructors typically engage in their own communities of practice to receive mentoring and emotional support. While didactic, one‐time trainings or workshops on topics such as mindfulness and managing challenging child behaviors have an important role, research demonstrates that experiential learning that occurs in the context of a relationship produces a greater impact (Brunsek et al., 2020; Sheridan et al., 2009).

The HiH approach offers educators a framework for understanding the purpose of children's emotional expressions and their role in supporting them through connection and non‐direction. It is meant to support educators of all experience levels, including those who already use one or more social‐emotional curricula or strategies in their daily work. Importantly, while HiH offers a (potentially novel) theory as well as some concrete strategies, it is intended to be used flexibly and integrated into educators’ existing repertoire of strategies. In the instances where educators feel that a response that prioritizes nondirective listening and connection is in contradiction with other strategies that they have been trained in, HiH is not meant to override or supersede those strategies. Rather, the intention is for educators to grow their reflective capacity in the mentoring calls and Listening Partnerships, and then bring a reflective stance to these decisions regarding how to respond to a child in the moment. Through reflection, educators may select from among their repertoire of strategies to decide how they want to respond, and then to consider how their response went for themselves and for the child. The intention is for educators to build a tendency to approach responding to children with intentionality. While they may increase their use of the HiH tools over time, more broadly, the training is intended to show educators how to use connection and listening as organizing principles in their interactions with children.

The intention for evaluating the Foundations Course, which was named a promising practice by the Association for Maternal and Child Health Programs (AMCHP, 2022), is to continue to refine the course and to prepare to scale it up to a larger audience over time. The research team is applying a rapid‐cycle, iterative, community‐engaged research model developed by the Frontiers of Innovation (FOI) program at the Center on the Developing Child at Harvard University. The research team is applying the FOI four‐phase evaluation process: (1) a feasibility study, (2) an early pilot study, (3) a late‐stage pilot study, and (4) a randomized controlled trial. An important emphasis of the research process is to work with participants as partners and experts whose feedback is valuable; through the research process, participants are co‐creators whose insights directly affect program development. The impetus is to integrate participants’ feedback into iterative improvements to the program. Rather than intervention science conducted by outside experts, the FOI model sees the value in organization‐conducted research done in partnership with service providers and those served. Bias is minimized through explicitly seeking information on what does and does not work, and for whom, while centering the expertise of participants.

The research team previously completed the feasibility study, in which a sample of 11 educators participated in the course and provided feedback. The goal was to obtain feedback on the course and its materials, and to critically examine what was working well and what needed improvement. The educators described feeling well supported through engaging in the mentoring calls and shared how they both learned and applied the five skills in their roles. They made suggestions for modifications to make the course more accessible and engaging for them that have subsequently been incorporated into the curriculum (e.g., increasing the length of the program, developing condensed materials for easier reference; Coleman et al., 2021).

The current study reports the results of the early pilot study, which represents the next step in establishing the evidence base for this course. The impetus for this study was to explore, with a larger sample, educators’ reactions to the revised course and their feedback for further improvement. Of particular interest was the extent to which the course led to changes in educators’ thoughts, feelings, and behaviors that could promote more attuned interactions with children as well as their own well‐being. To continue to refine the course, the research team also sought to understand educators’ feedback regarding course limitations, areas for improvement, and their ideas for changes to be made. Specifically, the study addressed the following research questions.

1. How do educators describe their experiences learning and applying the HiH connection‐based theory of emotional functioning and the role of listening?
2. How do educators describe their experiences learning and applying the five listening tools?
3. How do educators describe the impact of the HiH approach?
4. What feedback do educators have about the Foundations Course?

## 2 METHODS

In the Fall of 2022, the Foundations Course was piloted with 17 concurrent cohorts, each with four or five educators and led by a Certified HiH instructor. All participants were early care and education professionals who were invited to be part of the current study. Data collection consisted of weekly surveys, a post‐intervention interview, and pre‐, post‐, and 3‐month follow‐up surveys.

### 2.1 Procedures

Study procedures were approved by the Castleton University IRB. Early childhood education (ECE) staff were invited to participate in the early pilot study using a convenience snowball approach. A recruitment flier was posted on the HiH website with a form to be contacted for more information. In addition, study team members and HiH instructors (who live in many parts of the United States) disseminated information about the Foundations Course and this study broadly with their networks. In their outreach, they shared a recruitment handout with ECE staff and families with whom they had an existing relationship, inviting them to apply and to share the recruitment information with their own networks. Wherever possible, multiple educators from the same center were encouraged to apply so they could participate together.

The recruitment materials included a link for interested individuals to indicate their interest in the Foundations Course and in the study related to the course. Ultimately, 210 individuals applied. On the application survey, they reported their role in the ECE field, the ages of children they serve, their availability, their interest in participating in the pilot study, and more demographic information. Participants were selected by the study team and course designers. They planned to recruit 70 participants, hoping for a sample of 50 to complete the course and study and anticipating attrition. In reviewing applicants, they gave preference to (1) individuals in ECE positions with contact with students and/or staff who could benefit from the course and who were willing to participate in the study, (2) individuals working in Head Start or otherwise serving underserved communities and (3) groups of individuals from the same programs. The consenting process was thorough. In 30‐min consent calls, HiH research associates read the consent letter aloud to the potential participants and engaged them in dialogue about it. The study team placed particular emphasis on the consenting process because they were asking individuals to participate in both a course and a study, so it was important to ensure that they fully understood and freely consented to both components. Researchers explained to educators that their perspectives were valuable as the team worked to gain insight about the Foundations Course, including anything that could be improved.

Consented participants were assigned to cohorts of four or five people based on scheduling considerations. When there were multiple educators from the same program, they could opt to be placed in the same cohort or be separated. Directors were grouped together in their own cohort so that they could discuss their unique concerns on their mentoring calls, and to avoid the potential role conflicts that might present themselves if directors and their staff were grouped together. After the consenting process, there were 73 participants in 17 cohorts representing 21 states and 54 programs.

Before the first mentoring call, all participants were invited to complete the pre‐course survey, and after the final mentoring call, all were invited to complete the post‐course survey. During the eighth and final mentoring call, the interview data were collected. The interviewer was a member of the study team, and the Instructor was not present to avoid biasing educators’ responses. In March 2023, participants were invited to complete the 3‐month follow‐up survey.

### 2.2 The Hand in Hand Foundations Course for educators

Between October and December 2022, all cohorts engaged in the eight‐week course, which consists of weekly asynchronous didactic activities and weekly, hour‐long, synchronous group mentoring calls. The weekly didactic activities included readings and videos that were shared with participants via a web‐based platform. These resources introduced participants to the guiding theories of HiH approach as well as the five listening tools. Importantly, each listening tool was the focus of didactic activities for one week.

In the mentoring calls, the cohorts met with the Instructor. During this time, the Instructor facilitated discussion around each tool, providing individualized information and experience as well as appreciation and encouragement for participants’ experiments in using the tools. The educators could discuss their use of the tool taught that week, or any previously discussed tools, so educators had less cumulative exposure to the tools introduced later in the course (e.g., Playlistening). The calls also included time for peer‐to‐peer sharing and emotional support, based on the Listening Partnerships tool. The overall tone of the mentoring calls was intended to be safe and warm, and through modeling, the instructors were to demonstrate the values of listening and connection.

Instructors for the Foundations Course had all been previously certified as HiH instructors. The certification process requires a year‐long, 300‐h training culminating in a rigorous evaluation of their understanding, use, and communication of the HiH approach. All HiH instructors have access to a suite of professional supports, including their own mentoring calls, communities of practice, and additional trainings. The group of HiH instructors selected to teach the Foundations Course received additional course‐specific training and participated in mentoring concurrent with the Foundations Course.

### 2.3 Participants

While participants held various roles in their early childhood education centers, they will be referred to as educators for the sake of parsimony. There were 85 educators who were invited to participate in the study. Of those, 73 decided to participate after the informed consent process. Twelve educators attended three or fewer weekly calls and did not continue with the class or study (see Table 2).

**TABLE 2:** Number of weekly calls attended by educators.

Number of calls attended	0	1	2	3	4	5	6	7	8
Number of educators	3	1	5	3	2	4	11	14	30

*Note*: Educators who attended three or fewer calls were excluded from the analytic sample.

The analytic sample consisted of 61 educators. The majority of educators in the sample were White, English speaking, highly educated, and very experienced as early educators. See Table 3 for demographics.

**TABLE 3:** Participant demographic data, divided by enrollment and attendance.

	Enrolled and attended 4+ sessions (*n* = 61)	Enrolled and attended < 4 sessions (*n* = 11)	Did not enroll (*n* = 13)
**Language primarily spoken at home**	Frequency (percent)	Frequency (percent)	Frequency (percent)
English	55 (90.2%)	6 (54.5%)	1 (7.7%)
Spanish	3 (4.9%)	1 (9.1%)	1 (7.7%)
Urdu			1 (7.7%)
Missing	3 (4.9%)	4 (36.4%)	10 (76.9%)
**Race/ethnicity**			
African‐American or Black	7 (11.5%)	4 (36.4%)	
Asian	3 (4.9%)		1 (7.7%)
Hispanic or Latina/o/x	7 (11.5%)	1 (9.1%)	1 (7.7%)
White	38 (62.3%)	2 (18.2%)	1 (7.7%)
Biracial/Multiracial*	2 (3.3%)		
Prefer not to answer	2 (3.3%)		
Missing	2 (3.3%)	4 (36.4%)	10 (76.9%)
**Highest degree**			
Less than high school	0 (0%)		
Some high school	0 (0%)		
High school diploma or equivalency (GED)	2 (3.3%)	1 (9.1%)	
Some college	8 (13.1%)	1 (9.1%)	
Associate degree	6 (9.8%)	1 (9.1%)	1 (7.7%)
Bachelor's degree	21 (34.4%)	3 (27.3%)	
Master's degree	19 (31.1%)	1 (9.1%)	1 (7.7%)
Doctorate or Professional	1 (1.6%)		
Other	2 (3.3%)		1 (7.7%)
Missing	2 (3.3%)	4 (36.4%)	10 (76.9%)
**Age**			
18–24	1 (1.6%)	1 (9.1%)	
25–34	16 (26.2%)	1 (9.1%)	1 (7.7%)
35–44	18 (29.5%)	2 (18.2%)	2 (15.4%)
45–54	12 (19.7%)	2 (18.2%)	
55–64	7 (11.5%)	1 (9.1%)	
65 and over	5 (8.2%)		
Missing	2 (3.3%)	4 (36.4%)	10 (76.9%)
**Income**			
Less than $5000			1 (7.7%)
$15,000 to $29,999	2 (3.3%)	1 (9.1%)	
$30,000 to $44,999	12 (19.7%)	1 (9.1%)	
$45,000 to $59,999	7 (11.5%)		1 (7.7%)
$60,000 to $74,999	12 (19.7%)	1 (9.1%)	
Greater than $75,000	21 (34.4%)	4 (36.4%)	1 (7.7%)
Prefer not to answer	5 (8.2%)		
Missing	2 (3.3%)	4 (36.4%)	10 (76.9%)
**Student status**			
Full‐time	3 (4.9%)	1 (9.1%)	1 (7.7%)
Not a Student	48 (78.7%)	5 (45.5%)	2 (15.4%)
Part‐time	8 (13.1%)	1 (9.1%)	
Missing		4 (36.4%)	10 (76.9%)
**ECE setting**			
Center daycare	25 (41%)	6 (54.5%)	3 (23.1%)
Early head start	6 (9.8%)		
Head start	2 (3.3%)		
Home daycare	5 (8.2%)		
Preschool program that is part of a K‐12 school	9 (14.8%)	1 (9.1%)	
Kindergarten	3 (4.9%)		
State‐funded preschool	3 (4.9%)		
Other	5 (8.2%)		
Missing	3 (4.9%)	4 (36.4%)	10 (76.9%)
**Years as an early childhood educator**			
0–1 year	4 (3.3%)	2 (18.2%)	
2–3 years	7 (11.5%)	2 (18.2%)	
4–5 years	7 (11.5%)		
6–7 years	10 (16.4%)		1 (7.7%)
8–9 years	1 (1.6%)	1 (9.1%)	
10–12 years	4 (3.3%)		
13–15 years	4 (3.3%)		1 (7.7%)
More than 16 years	22 (36.1%)	2 (18.2%)	
Missing	2 (3.3%)	4 (36.4%)	10 (76.9%)
**ECE role** ^a^			
Lead teacher	35 (57%)	1 (9%)	3 (23%)
Director	12 (20%)	3 (27%)	1 (8%)
Assistant teacher or other educator	12 (20%)	2 18%)	
Specialist	7 (11%)		
Owner	2 (3%)	1 (9%)	
Other	1 (2%)		
Missing	2 (3%)	5 45%)	10 (77%)
**Familiarity with Hand in Hand before course**			
No familiarity	18 (29.5%)	3 (27.3%)	1 (7.7%)
Some familiarity through website and/or conversations with other people	23 (37.7%)	3 (27.3%)	1 (7.7%)
Familiarity through 1‐ or 2‐day training provided by Hand in Hand Instructor(s)	1 (1.6%)		1 (7.7%)
Familiarity through an online or in‐person course with Hand in Hand	5 (8.2%)		
Other	12 (19.7%)	1 (9.1%)	
Missing	2 (3.3%)	4 (36.4%)	10 (76.9%)

^a^ Totals exceed 100% because many respondents held multiple roles.

To further understand the participants, it is important to highlight their prior training experiences. First, perhaps related to the fact that this pilot study used a convenience sampling approach, many had some degree of prior exposure to HiH, despite the fact that the Foundations Course is an introductory level course that does not require any prior knowledge of the HiH theory or tools. While a significant proportion of this prior exposure was fairly superficial (e.g., review of the HiH website) or was not described, six of the educators who enrolled in the study had received prior training from HiH. While participants may have engaged in trainings that included similar content, those trainings either lacked the experiential component of the mentoring calls with a cohort of peers, were significantly shorter, and/or were developed for parents and did not incorporate any considerations for the ECE setting. Given the small sample size, comparisons between those with and without prior exposure to HiH were not feasible. Educators also shared in an open‐ended question about the educational philosophy and/or social‐emotional learning curriculum that their program uses, as well as any others in which they have received training. Educators’ responses varied widely and included several dozen unique entries. Educators listed educational philosophies and curricula such as Montessori, Second Step, Responsive Classroom, and High‐Scope. They also listed other approaches in which they had been trained, including Pyramid Model, Conscious Discipline, Incredible Years, RIE, Zones of Regulation, and Touchpoints. Educators’ responses, on the whole, show a high level of exposure to professional development related to social‐emotional topics.

Attendance to mentoring calls was variable; out of 8 total, the average number of sessions attended was 6.2, and the median was 8. Demographic data gathered from those who attended three or fewer calls were reviewed to investigate whether there were patterns in retention and accessibility of the course. The considerable amount of missing data made it challenging to draw valid conclusions. Of those who enrolled, a higher proportion of Black/African American educators did not complete the course compared to other racial/ethnic groups. However, we do not have data on reasons for non‐completion, and the group sizes were too small to support statistical comparisons. The results in this manuscript focus on data from the 61 educators who attended at least half of the calls, unless otherwise noted.

### 2.4 Measures

#### 2.4.1 Surveys

All surveys were sent via Qualtrics, a web‐based survey platform. Participants were given a small incentive for survey completion ($10 for the Pre‐Course Survey, $10 for each Weekly Survey, and $25 for the 3‐Month Follow‐Up Survey). The surveys and interview protocol were designed by the research team to specifically address perceptions of the Foundations Course. Participants could skip items that they chose not to answer.

**Pre‐Course Survey**. This survey included demographics, background information on educators’ current and prior positions, and interest in participating in the Foundations Course. In this survey, educators indicated what, if any, prior familiarity they had with HiH.

**Post‐Course Survey**. This survey asked educators to provide feedback on the course as a whole, and to rate the usefulness of each listening tool, the mentoring calls, and the didactic materials. It also asked educators to rate changes in their confidence in nine common classroom situations (e.g., “supporting children to participate in classroom routines”) on a 10‐point scale from “much less confident” to “much more confident.” They were also asked to rate their understanding of each of the five listening tools. Throughout, participants were invited to add qualitative responses to explain their ratings. For participants who did not complete the pre‐course survey, the demographic information was collected in the post‐course survey.

**Three‐Month Follow‐Up Survey**. The follow‐up survey asked educators whether they used each of the five tools and if so, to describe their experiences using it. It also included a series of open‐ended reflection questions about changes for them and their classes since the course; examples of using the five tools; suggestions for improving the course.

#### 2.4.2 Interview

To gather qualitative data, the research team designed a short, semi‐structured interview protocol which was administered and recorded during the final mentoring call for each cohort. In the 45‐min interview, each of the four or five participants were asked the same questions. The interviewer managed time so that all participants had equal opportunities to speak. All participants were informed that they had a defined amount of time to respond. The questions were: “How was your attitude about your own or other's emotions and behaviors influenced or not influenced since you took this class?” and “If you used/incorporated any of the tools, how was it? If you did not, please share more about that.” Time permitting, the interviewers asked follow‐up questions for each participant to better understand the scenarios that they were describing and/or their barriers to using the skills. While timed responses may seem limiting, they were consistent with the course format, thereby providing familiarity and continuity. Specifically, the tool of Listening Partnerships, which the educators practiced each week on the mentoring calls, involved sharing for a set amount of time. The interviewers were HiH instructors, but were unknown to the participants because participants were never interviewed by the Instructor that led their mentoring calls. The interviewers were all female, held graduate degrees, and had prior research experience.

### 2.5 Data analysis

#### 2.5.1 Quantitative data analysis

Survey data were exported from Qualtrics into SPSS. Descriptive statistics were used to summarize the data.

#### 2.5.2 Qualitative data analysis

Interviews were automatically transcribed by the Trint software, and then edited for accuracy by a researcher, and then checked by a second researcher. The interviews were then uploaded into the qualitative analysis software, Dedoose. A priori codes derived from the results of the feasibility study (Coleman et al., 2021) were used for the initial round of coding; these codes included “challenges” and “positives” for each of the listening tools to help ensure the analytic process did not overly emphasize one or the other. A lead coder led a team of five in line‐by‐line coding of the interview data. To reduce bias during the coding process, the team did not discuss analysis or implications of the data, but rather focused only on accurate coding. Coders met regularly to discuss coding procedures. The lead coder reviewed 20% of the interviews and the rest were done independently, with any questions being discussed as resolved as a group. These codes were then consolidated into broader categories by the coding team. A second consolidation was done to refine this and reduce redundancy where applicable. Four members of the research team then met to review all codes, generate themes, and then consolidate the codes into those themes.

Qualitative analysis was also conducted for qualitative survey items. Survey coding took place after interview coding. Whereas the interview coding used a deductive and inductive approach to refine the codes and themes, and survey coding used a deductive approach, using only the established codes and themes from the extensive interview coding. This reflects the fact that the interview data were much richer and more nuanced than the survey data and no new constructs were introduced in the surveys, so the research team felt confident relying on these themes and codes. Because the coding team had already reached saturation for two themes during interview coding, those were omitted from survey coding. A coding team of two researchers independently assigned themes to the qualitative survey responses, meeting to resolve discrepancies until all codes were finalized. The coders for the interview and survey data were all well‐versed in reflective practice, which enables one to step back and examine their reactions and motivations. In this way, they were well‐positioned to set aside preconceived notions during the coding process.

#### 2.5.3 Data integration

In this convergent mixed‐methods design (Creswell & Plano Clark, 2011), data integration happened during the analysis and priority was given to the qualitative data elements. Until that point, all quantitative and qualitative elements were conducted separately. Furthermore, to privilege the voices of participants, thematic analysis occurred before analysis of quantitative data. When thematic analysis was complete, the research team reviewed the themes alongside the survey data to look for consistencies and discrepancies. In the results section below, each research question has qualitative and quantitative data represented, though the richer qualitative data is emphasized. Insights derived from comparing qualitative and quantitative data are described.

## 3 RESULTS

The results are organized by research question. The qualitative data analysis resulted in four themes, each with corresponding codes (see Table 4). Each theme and most of the codes will be described in the corresponding research question. Two codes (connection first and tools are interconnected) had overlapping content with other codes and were omitted for parsimony. While the qualitative data yielded the greatest insights into the Foundations Course, the related survey findings are also included below as they pertain to each research question. A summary of the benefits and challenges identified in the qualitative data can be found in Appendix B.

**TABLE 4:** Final themes and codes for qualitative coding.

Themes	Codes
Shift in lens: The power of listening and the function of emotions	Power of listening Emotional expression has a function Connection first
Benefits and challenges of using the HiH listening tools	Listening tools are valuable: “One of the best tools” Learning takes time and support: “I'm still thinking about how to use the tools”
Benefits for ECE communities	Teachers feel better Relationship benefits with children, families, and colleagues
Course revisions needed to better serve educators	Challenges to learning Challenges to implementation Tools are interconnected

### 3.1 RQ1: How do educators describe their experiences learning and applying the HiH connection‐based theory of emotional functioning and role of listening?

The Foundations Course teaches educators the HiH approach. While the HiH approach is operationalized in the five listening tools, they are all undergirded by the theory that emotions are functional, and that attuned listening builds connection and fosters the conditions for processing emotional experiences, which in turn promote emotional flexibility and learning readiness. While preparing educators to use the individual listening skills is a major component of the course, it is important that they first learn the principles guiding those tools. One of the four key themes from the qualitative analysis of educator feedback addresses this research question. The coders named this theme: **“Shift in Lens—The Power of Listening and The Function of Emotions.”** Overall, most educators shared that the course helped them cultivate a new perspective. They described shifts in their understanding of—and responses to—children's emotions and behaviors. Educators also reported shifts in understanding and processing their own and other adults’ emotions. Broadly, educators described an enhanced appreciation for the power of listening, connection, and emotional expression.

**The Power of Listening First**. Educators reported greater understanding that “something as simple as just listening can be so powerful” in their interactions with children and with peers. To foster experiential learning, they learned what it felt like to be truly listened to during the weekly mentoring calls. On the calls, they shared about their experiences as educators with the Instructor as well as their peers. In these interactions, they received recognition and validation for the challenges they faced in their caregiving roles and their associated “moments of great rewards and moments of struggle.”

In parallel, many educators began taking a “listening first” approach to their interactions with children, rather than responding to children's heightened emotions with a goal of fixing or distracting. As a number of participants articulated, “[s]ome of the tools in Hand in Hand reminded me…not to try to label something, but just to notice it” and try “not to be the problem solver but a listener and less reactive.” By “being present, and providing warm connection and reassurance, the child can resolve the emotions themselves.” Overall, some educators shared that “[m]y initial responses [to emotional outbursts] have changed significantly.”

**Emotional Expression has a Function**. Educators described a shift in their understanding of the value of experiencing and expressing emotions. They shared that the course helped them recognize that emotional expression has an important function. Educators noted that the HiH approach is “pretty revolutionary,” suggesting that adults stay with children so they can safely express emotions, rather than seek to control, redirect, or calm their emotions. For many educators, this perspective required a mindset shift. One educator noted that she previously felt like children display “intense emotions and those moments with a lot of feelings” because they “don't know any better,” but now sees that “they're trying to communicate to me. This is what they need help with…” As another noted, “I see now and I understand better…the healing power of the emotions, the release of emotions.” They also witnessed the payoff of creating space for children's emotions. By “…[r]eally allowing the child to move through the emotional experience,” educators learned and witnessed that children were able to work through their emotions and feel more connected to the educator; it …was the kind of soothing balm that they needed.”

The HiH approach fundamentally changes the educator's role in moments of heightened emotion, and some reported that this reduced some of the pressure and strain they felt. One described going from feeling like “I don't know what to do” in response to children's emotions to feeling that “I don't need to know. They probably need connection…being reminded to see it as …a cry for help and not having to worry about why they're doing what they're doing or what they need. I can just be moving close and I don't even have to say anything about it and just…be with them and give them my full attention and my love, and they'll figure it out.” One described changing from having a “busy energy” to being more “relaxed” when children have emotions. “Now that I'm understanding that there's a process, you get it, you witness it, support it, and then it resolves itself. It really does.” This approach differs from some of the ways they had previously been taught to respond to children's emotions and behaviors. For example, “[a] lot of the social emotional curriculum that we use at school revolves around labeling feelings and just some sort of resolution. And so this course really kind of made me take a pause before anything in those situations, even when it came to myself, just giving myself and the students space to just feel and go through whatever the situation is, even without labeling the emotions that are coming up and just giving space to just like cry or, you know, be upset.”

Even educators that had previously been accepting of or receptive to emotional expression reported that they were better able to recognize the healing function of emotions. “…before this class, I was always open to emotions. But, what I didn't know or think about was that emotions are our brains’ way of healing itself. And the biggest change for me in my parenting with emotions has been realizing that when my children are having an emotional upset, it's their brain's way of asking me for help reconnecting.” It was helpful for the educators to reflect on the function of emotions for themselves as well as for children. Educators affirmed that, “teachers and parents need to have their own way to emote and release feelings in order to be effective instructors and loving parents.” This can be especially true when interactions with children trigger past emotional experiences. Educators appreciated learning that there are a range of ways that they can express emotions to relieve tension, especially within the confines of their workday.

Results from the survey data indicate that educators’ shift in perspective may endure over time. At the 3‐month follow‐up time point, 77.6% of educators reported that their thinking about children's emotions had changed because of the course. In addition to describing a mindset shift immediately after the course, most of them seemed to sustain this new perspective over time, though about a quarter did not.

### 3.2 RQ2: How do educators describe their experiences learning and applying the five listening tools?

The Foundations Course taught the educators the theories described above as well as five listening tools. The five listening tools are: Listening Partnerships, Staylistening, Setting Limits, Special Time, and Playlistening (see Table 1). Each tool gives a concrete way to enact the value of listening, establish and strengthe the connection between adult and child, and allow for emotional expression. As summarized in the theme called “**Benefits and Challenges of Using the HiH Listening Tools**,” educators in the course described adopting these (often new) skills in their work with children and seeing a positive impact on their relationships with children. There were also challenges to implementing the skills in the classroom, which include logistical challenges as well as challenges in learning and adopting new practices.

**Listening Tools are Valuable: “One of the Best Tools.”** Many educators found the set of tools to be valuable. This positive perception of the tools was summarized by one educator: “[t]hese tools are rooted in what comes easiest: loving children. The program highlights ways to incorporate everything that happens in early childhood classrooms into a joyful dance. The simplicity of the tools is understandable and sustainable.” The survey data aligns with the positive perception described in the interviews. When surveyed at the end of the course, many educators rated the value of the tools and the usefulness of the Foundations Course highly, as shown in Table 5.

**TABLE 5:** Educator final survey ratings.

	*N*	Min.	Max.	Mean	Std. Dev.	Median
How valuable are the Hand in Hand tools to you?^a^	47	5	10	9.43	1.14	10
Do you think these tools could be used effectively in your specific program or setting?^b^	45	4	10	8.93	1.41	10

^a^ Response options ranged from 1 to 10, where 1 = not at all useful and 10 = very useful.

^b^ Response options ranged from 1 to 10, where 1 = not at all and 10 = very much so/a lot.

In the interviews and surveys, educators shared their experiences using each of the five listening tools. A brief summary of their reactions to each tool is provided below. Educators shared their experiences using each of the five listening tools, including any challenges they experienced with the specific tool. Overall, challenges implementing the tools are also described in the subsequent code, **Learning takes time and support: “I'm still thinking about how to use the tools.”**

***Listening Partnerships*** are structured times for adults to provide support for each other by taking equal turns listening to each other without interrupting or giving advice. Educators practiced Listening Partnerships on the mentoring calls and were encouraged to develop a peer‐to‐peer Listening Partnership outside of the course as well. The educators reported appreciating the opportunity to share their feelings in a safe, supportive setting, though some expressed feeling awkward or avoidant in the beginning. Educators described how it was “really powerful” and nurturing to express their thoughts and feelings without being judged, and how expressing feelings in this context feels like “a weight lifted off the shoulder.” After experiencing the benefit of receiving undivided attention and caring from someone else, educators described the ways in which it helped them offer this listening to children, colleagues, and parents. In so doing, “…I really did [see the child] because I stayed quiet. I stayed quiet.” Some educators struggled to implement Listening Partnerships at work without the scaffolding provided during the mentoring calls. The barriers included identifying the right person, having enough time, knowing how to initiate a partnership, and feeling awkward. Consequently, some educators did not end up using the tool outside of the mentoring calls.

***Staylistening*** is an approach through which adults offer children support during emotional moments. The adult listens to the child's feelings and communicates their presence, attention, and care, while setting limits on off‐track behavior. Educators reported using Staylistening very often (“all the time,” “usually multiple times a day”). In these moments, they learn more about the children: “It just was another window into her, into her and her life. That was fascinating…” In response, children may express affection towards educators: “he says, ‘I love you, Mrs. [name] I love you, I love you.’… He knows I care about him.” Educators began “noticing a lot better behavior after the upsets” when they are “not trying to talk them out of their feelings or like, you know, the impulses… Just to let them be…” Overall “[t]here's not as much crying or seeking adult attention because they got it. They got the attention when they needed it most.” Educators reported few barriers to using Staylistening other than a lack of time and staff. To fully attend to a child during Staylistening, there must be a second educator present to supervise the other children in the classroom and to maintain the daily routine.

***Setting Limits*** is when a calm adult responds to a child's off‐track behavior by setting limits on the behavior, while providing warm, attuned listening to their feelings (i.e., Staylistening) before and after setting the limit. Many educators found Setting Limits to be simpler to implement than other techniques that they could use in response to challenging behavior. One educator noted: “…setting limits is great. I like how it's gentle and it's few words. And it works.” Educators reported a feeling of relief in shifting the focus from reprimand to rebuilding connection: “it's like an attitude change about setting limits, about not yelling across the classroom.” They reported feeling “gentle,” “calmer,” and “not elevated.” This approach allowed daily limit‐setting to be a warmer process, which again brought relief to the educators. They did not describe any barriers to using the Setting Limits tool. Setting Limits is an aspect of Staylistening, and educators did note challenges with Staylistening. It is possible that they did not note the same challenges with Setting Limits because they did not understand its connection to Staylistening.

***Special Time*** is one‐on‐one, child‐led playtime. Overall, educators who tried Special Time in their classroom shared that the tool had benefits for children and for themselves, but could be challenging to implement. After taking this one‐on‐one time, “…I feel closer to the kids. They feel closer to me” which can help “turn the corner in terms of a relationship with a kid.” Educators found that Special Time has a beneficial effect on themselves, noting, “also it changes my behavior and how I act toward certain situations in the class. So that's been a plus.” Following the child's lead in the play was fun for teachers, for whom it provided “a really wonderful moment for me to not be in charge” and a break from “surveilling and going in when there's a problem,” but at the same time, educators also reported that it was challenging to stay in the “follower” position, without scaffolding or leading the play. They gave examples of times when they would naturally give suggestions or ask questions in the play, and needed “more practice… to make sure that I'm not being the one that's like telling him what to do.”

However, Special Time can be logistically challenging in the classroom environment, and one educator noted that, of the five tools, Special Time was the most challenging to implement.

Ideally, Special Time is proactively incorporated into the classroom routine, for example, educators may schedule in short periods of 5 min during the day when they will offer Special Time, scheduling each child for this time once a week. Support from co‐teachers and the larger program is especially helpful during these times because educators have to make sure other children are being attended to. Also, it is hard to institute Special Time reactively in a moment if “they all want the attention” at the same time. Having this schedule visible to the children so they can see that they will each receive Special Time at some point helps them tolerate and allow space for the times that other children are receiving Special Time.

***Playlistening***. Overall, educators who tried Playlistening shared that it helped ease tensions for children and helped children “work through their stuff,” that it is fun to do for both children and teachers, and that it can be “used in the moment” and “in transitions.” Some educators shared that this tool was “easy” and aligned with their play‐based orientation, or even something they were already doing before the course. Others shared that being playful was more difficult and “it's a little awkward at first, but just kind of relax into that and, and play like that, and you get a lot of mileage out of it.” Educators gave examples of how Playlistening helped them cultivate a more playful approach throughout the day (e.g., transitions, meals, rest/nap time, going to the bathroom, getting dressed, and during children's play) while encouraging on‐track child behavior. This can help children realize that “the adults are here to help you and we're here to support you. And we're not just these, like, rule‐enforcers that are stopping you all the time.” Educators also shared that taking the less powerful role helped build relationships and ease tensions. One educator noted that Playlistening “helps me as much as it helps the child.”

**Learning takes time and support: “I'm still thinking about how to use the tools.”** The Foundations Course is eight weeks long; each week, on top of their jobs, educators attended one mentoring call and reviewed a variety of materials. In this space, they were taught the HiH theory as well as the five listening skills. Learning takes repetition and practice, and there were limits to how much could be learned and absorbed in the time allotted. It is impressive that, 3 months after the course, all but one of the 49 educators (98%) who completed the follow‐up survey reported having used at least one tool in the past 3 months. Yet in their qualitative comments in the final and 3‐month post surveys, it was striking how seldom educators described using specific tools. Rather, the majority of their remarks about content from the course referred to the theories, sharing examples of how the emphasis on listening and connection remained with them after the course and informed their interactions with children. When specifically asked about their use of each tool in the 3‐month post survey, their responses often demonstrated incomplete understanding of the tools, though the fidelity of their implementation was not directly assessed. Overall, participants responded in ways that showed a clear understanding of the theory and made fewer references to using the individual tools.

In light of this trend, it was surprising to note that most educators rated their understanding of each tool highly. By the end of the course, most educators reported that they understood the five tools well. Table 6 shows educators’ ratings of their own understanding of each tool on a scale from 1 (low understanding) to 5 (high understanding). The average scores for each tool were all over 4 on the 5‐point scale, with the lowest ratings for Playlistening and setting limits.

**TABLE 6:** Educator self‐ratings on final survey.

	*N*	Min.	Max.	Mean	Std. Dev.	Median
Understanding Special Time	44	3	5	4.57	.70	5
Understanding Staylistening	45	2	5	4.47	.79	5
Understanding Playlistening	45	2	5	4.04	.98	4
Understanding Setting Limits	45	2	5	4.11	.91	4
Understanding Listening Partnerships	44	3	5	4.59	.66	5

### 3.3 RQ3: How do educators describe the impact of the HiH approach?

After learning the HiH approach and beginning to implement the five listening tools, educators began to see the impact of these new ways of connecting with children. In the theme **“Benefits for ECE Communities,”** coders identified that educators reported benefits for themselves, as well as children, families, and colleagues.

**Teachers feel better**. Educators reported that “feeling better trained, better informed, and more confident was powerful.” They described being “calmer,” “warmer,” and “more compassionate” with children and “less rigid and reactive” with children and with the expectations they placed on themselves. They found conflicts between children in their settings “not as stressful” and remarked that they were better able to maintain a “sense of calm in most any storm, both personally and professionally with children and with parents.” An educator described having new resources to handle aggression in the classroom as “enlightening” and a “relief.”

Educators also reported increased self‐awareness and more meaningful connections. One described noticing their emotions more quickly and, as a result, gaining insight into the limits that they needed to set to protect their own wellbeing. Put simply by an educator in the study, “you can't give what you didn't get.” Another educator described the unexpected way in which they benefitted: “I am more grounded and allow others to see and hear me. This is a surprise to me, as I thought I would be doing all the listening! The more I am able to fully listen and be present, peers and children seem to hear me.” Furthermore, educators were encouraged to notice the ways in which their own early experiences might be affecting their reactions to children, providing an opportunity for self‐reflection. For example, one educator noted their desire to “be seen” in the classroom, noting that they in turn “have a really hard time giving that to these children.” By reflecting on the impact of their emotions and early experiences on their interactions with children, educators could move forward with greater intentionality.

On the final survey, educators were asked to report on whether their confidence in managing a series of emotionally salient classroom situations had changed compared to before the course. Over half of all respondents reported increased confidence for each item.

**Relationship benefits with children, families, and colleagues**. Educators described how the impact of the course on themselves led to stronger connections with children, families, and colleagues. Educators credit the HiH approach with leading to greater closeness with children; “to see how much more they give hugs, how they love you. Lean into you after those big feelings when you just let them have them and and are there. That's been huge for me.” Some noted that the HiH approach was particularly impactful for children who have “seemed to really need it.” Examples included both children who need an opportunity to express emotions that they otherwise might keep contained, as well as children for whom other social‐emotional learning approaches have not been effective. One educator noticed that, after using the connection‐based tools, children may show more willingness to participate in classroom activities and cooperate with directions. Compared to before the course, 81.3% of educators reported that they have more positive interactions with children, and 56.3% reported that they have fewer conflictual interactions with children.

Educators also described sharing the tools with parents, supplementing their usual parent education approach with the listening tools. According to one educator, the relative simplicity of the course made it easier to share concepts with families. Another educator noted that the HiH approach helped them talk with parents about their children's emotions. “This has given me the tool to be able to…discuss that better with parents. Right. To be able to say, well, let's step back, let's support this child. Let's let them have their feelings.” Furthermore, educators were modeling these skills for parents.

“We really try to redirect those big feelings all day long with kids, and we're really kind of taught to do that…But when you really start getting into how transformative it is for them to express those feelings and get them out and not redirect them and distract them and just have them push it down all day, that's pretty revolutionary for a classroom. I think that's a really different way to manage your classroom. And to have parents see you do that and to be modeling that for parents so that they see what happens when you do that so that they're also not trying to just avoid tantrums all day long. They see the benefit of allowing the expression of those feelings and how much closeness it creates and how much safety it creates for those children and how they really come back into the sunshine after they get to offload… “

Some educators reported noticing changes in parents’ usual responses based on discussing and/or modeling the HiH approach. Educators shared stories of times when parents used the listening skills as recommended by the educator, resulting in emotionally supportive interactions at home and improved classroom interactions. Finally, some educators noticed that taking the Foundations course had changed their attitude towards the parents and families in their community: “I also noticed that in this class I'm much more generous towards the parents in my attitude.”

The Foundations course also supported relationships among teaching teams. Educators shared that they felt more connected to their peers, both in terms of understanding their perspectives and being ready to help one another. Multiple educators stated that it was useful to attend the mentoring calls with their co‐teachers, saying they are now “definitely on the same page a lot more in terms of how to handle certain situations.” The greater connection and understanding among teaching teams “…is reflected in the classroom when we're teaching together. Overall, the Foundations course supported educators’ relationships with children, families, and other educators, as well as enhanced relationships in the personal domains of educators’ own families and communities.

### 3.4 RQ4: What feedback do educators have about the foundations course?

As described above, educators had a largely positive reaction to the theory and the tools taught in the course. Nevertheless, they also described barriers to learning the content and implementing it in the classroom. The challenges they face suggest potential revisions to the course, which are summarized in the theme **“Needed Course Revisions to Better Serve ECE Communities.”**

**Challenges to learning**. Educators expressed some challenges to learning the HiH approach and made suggestions for ways that HiH could better support them to absorb the information presented. Many participants stated that they felt like the course was too short. They described feeling like it ended before they fully understood the tools and how to implement them. Educators suggested some form of continued exposure to the materials, including a longer course, monthly booster sessions, ongoing contact with the mentoring call group, and regular emails from HiH. Relatedly, some educators wanted more opportunities to practice the tools, both during and between mentoring calls. Other educators suggested revisions to the learning materials that would make them more applicable to their settings. For example, several educators commented that the materials depicting a listening tool being used in a home setting were less helpful because it was difficult to generalize to the classroom context. In addition, some educators noted a need for more materials demonstrating how adults use the tools with infants and toddlers. The differences in setting and child age are significant when it comes to giving useful examples of implementing these skills, and there is a clear need for more tailored examples. Educators also requested more concise resources (e.g., a handout) for each tool that they could refer to. Finally, educators described a lack of sufficient time to devote to the course.

**Challenges to Implementation**. Educators described a range of challenges in bringing the tools into their classrooms. Some challenges pertained to the other staff in their centers. First, in order to use the tools that required one‐on‐one attention, they needed a co‐teacher to supervise the other children, but they did not always have one. Also, a common barrier was a lack of buy‐in from other teaching staff; if the director and/or co‐teachers were not trained in HiH, they might not support the educator in implementing new tools. Sometimes the other staff might be uncomfortable with the HiH tools, especially if they contrast with their current practices pertaining to emotions. Educators requested resources (e.g., scripts) for describing the tools to their colleagues as well as to parents to seek shared understanding of these connection‐based strategies and recommended training whole centers at a time. Another implementation barrier was a lack of time in the daily schedule to use some of the more open‐ended tools. There are challenges to changing the status quo, especially in ECE settings characterized by delineated schedules, behavior management strategies, and staff ratios.

Even with these challenges, most participants ended the course with a favorable impression of the content. This was evident in their high ratings of the value of the course and the likelihood that they would recommend it (see Table 7).

**TABLE 7:** Educator final survey ratings.

	*N*	Min.	Max.	Mean	Std. Dev.	Median
How valuable was the Hand in Hand's Foundations Course for Early Childhood Educators class to you?^a^	47	6	10	9.23	1.17	10
I would recommend this program to other early care and education professionals (administrators, other allied professionals, home visitors, classroom teachers, mental health consultants, parents).^b^	45	5	10	9.44	1.04	10

^a^ Response options ranged from 1–10, where 1 = Not at all valuable and 10 = Very valuable.

^b^ Response options ranged from 1–20, where 1 = Strongly disagree and 10 = Strongly agree.

## 4 DISCUSSION

Educators in ECE settings are the cornerstone of high‐quality learning that supports child development across domains, and their interactions with the children in their care contributes to early relational health, whereby the health and neurodevelopment of young children (as well as the educators themselves) are advanced through their dynamic interactions. Despite their vital role in society, the precarity of educators in ECE has been well‐documented. In response to these challenges, the HiH Foundations Course has been developed and refined over the course of the past few decades to provide educators with a sustainable suite of tools to: (1) establish and nurture connections with the children, families, and colleagues through emotionally‐responsive practices; and (2) engage in reflective practices that build capacities for educators to care for themselves and others. By improving educator well‐being and equipping educators with tools to confidently accompany children as they experience emotions, the ultimate goal is to improve early relational health. This mixed‐methods pilot study investigated how educators from different ECE settings around the US perceived the Foundations Course. Specifically, it investigated how educators made sense of the theories and tools, the impact of these theories and tools on educators; and the potential improvements to the course itself.

The results of the study indicated that educators found the connection‐based theory to be resonant and transformative, with implications for their own well‐being as well as their relationships with children, families, and colleagues in their ECE settings. Strikingly, approximately three‐quarters of the participants reported that their views about emotional expression by children and adults had changed as a result of learning about HiH theoretical approaches and tools. The contention that a shift in approaches to understanding emotion‐having was “pretty revolutionary” reverberated throughout the findings. Three months after the Foundations Course, educators were still focused on the transformation of their own thinking about emotional expression, and the impact this change in thinking and approach was having on classroom behavioral dynamics, relationships with families, and relationships with other adult team members.

It is important to note that the HiH's work with ECE providers on the Foundations Course is closely aligned and situated within related approaches to build nurturing and responsive relationships in early childhood classrooms, best exemplified by approaches such as the Pyramid Model (Hemmeter et al., 2022). The suite of approaches associated with creating responsive classrooms center these practices as universal strategies to build early learning classroom cultures of kindness and connection in which positive identities are promoted and all children and families feel recognized, particularly those from minoritized racial and ethnic groups. What differentiates HiH approaches from these aligned efforts–and what is implicit in the findings from this study– is the creation of the space for individual and community reflection that truly makes room for and builds tolerance for even intense emotion‐having in children and adults. Once this space for deep reflection is created, there could be profound implications for the emotional regulation and well‐being of educators who are caring for young children, as suggested by the results of this mixed methods study. Future work can delve deeper into implications of the transformation of ECE provider conceptualizations about emotion‐having on classroom management, and more broadly on child and adult well‐being.

It would be remiss to discuss these results without noting that the course and its pilot study unfolded within the reality and messiness of the “post” COVID‐19 pandemic classrooms. At the time of the study, infants, toddlers, and young children born during the isolation of the pandemic years entered formal ECE settings for the first time. Recent research suggests that young children's social‐emotional development was disrupted during the pandemic (Kuehn et al., 2024), adult stressors increased in ways that affected their caregiving (RAPID Survey Project, 2024), and the ECE field experienced massive disruptions (e.g., closures, staffing issues, and social distancing, and distance learning) that affected educators well‐being (Weiland et al., 2021). The promise of the Foundations Course as a support for educator well‐being and early relational health, as demonstrated by this sample of educators operating at a uniquely challenging time, is notable and ripe for further research and policy attention.

While many educators described internalizing the value of leveraging emotional expression—the heart of the HiH approach—some educators struggled to fully grasp all five listening tools and to feel ready to implement them in classrooms after just an eight‐week course. This finding is unsurprising given the complexity of implementing interventions in real‐world educational settings. The findings from this study also demonstrate that educators reflected more on theory than on specific tools, and when they did reflect on specific tools, it was on the ones most closely linked to the theory (Staylistening and Listening Partnerships). Adult learners seemed to need more time and support to really use the listening tools, especially the tools covered at the end of the course. The immediate ECE program context could have been a barrier to sustainable implementation. Several participants reported on a lack of time or staff to implement HiH listening tools. Consistent with other research, participants reported that time constraints were particularly salient for Special Time, since this tool requires 1:1 time with children, and cannot be implemented as a multi‐child or whole class intervention (Cooper et al., 2024). Educators’ perceived challenges with the Foundations Course mirror challenges previously reported in the field; prior research shows that educators prioritize the promotion of social‐emotional development in their work with young children, yet feel limited by time constraints, group size, and their own confidence implementing strategies (Blewitt et al., 2021). These findings suggest that future evaluations of the HiH approach should focus more squarely on dosage, adult learning principles, and the balance of implementation fidelity with appropriate flexibility (Han & Weiss, 2005).

### 4.1 Implications for practice

Although participants in this study experienced challenges in fully implementing the HiH approaches, they helped shape potential improvements or alternatives for the course. First, this course is intended to develop familiarity—not expertise—with the HiH approach, though it seems that participants may have needed more support to learn and practice the tools in the ECE setting. This support could take multiple forms, including: a longer course, “booster sessions,” an in‐person component, and/or live observations. Future practice of training all staff center‐wide is indicated to pursue a “culture of connection” and to support sustained implementation of the HiH approach on the organizational and systems level, reducing the onus of implementation for individual educators. The engagement of entire ECE centers would allow interdisciplinary teams of educators, staff, related service providers, leaders, and families to coalesce into learning communities that can wrap support around children and one another. Indeed, the literature indicates that professional development may be more impactful when staff from the same center participate together (Zaslow et al., 2010), and that leaders play a crucial role in creating climates that welcome innovative practices, are ready for change, and support the process of implementation (Lyon et al., 2018).

### 4.2 Implications for research

Based on the results of this study, and in alignment with the four‐phase FOI evaluation process, the research team is beginning to formulate plans for future empirical investigations into the Foundations Course. Broadly, the team plans to evaluate the course with increasingly rigorous methods, including random sampling, comparison or control groups, and statistical controls. Future studies will also incorporate additional constructs of interest as mediators (e.g., the alliance between the educator and Instructor, the ECE setting type) and outcomes (e.g., educator‐child interactions) as well as more quantitative and observational measures. To do so may involve measurement development efforts given the uniqueness of the connection‐based theory informing the HiH approach. Building on both the findings from this early pilot study, and decades of implementing the Foundations Course to organizational teams outside of this study, the team plans to evaluate a whole‐center implementation of the course and to study the role of leadership engagement and organizational support for implementation.

### 4.3 Implications for policy

Structural changes that have long plagued the field of ECE have intensified in recent years, particularly as ECE staff shortages and funding shortfalls have become more pronounced as pandemic‐era stabilization funding has ended (NAEYC, 2024). At a time when educators are facing declines in workplace conditions and serious challenges to their health and well‐being, it is more important than ever to focus on educator well‐being and mental health in sustainable ways. Early childhood systems of care can work collaboratively to ensure that young children and their caregivers–including educators and family members– have access to high‐quality resources that equitably and sustainably support adult and child mental health and social‐emotional functioning. Recent policy guidance released by the US Department of Health and Human Services and the US Department of Education stresses the priority of improving early childhood mental health and social‐emotional development, in part by addressing workforce wellness (Administration for Children and Families, 2024). Additionally, at the time of this writing, the Head Start Performance Standards had just been revised to integrate and elevate mental health supports that promote staff and family well‐being, in addition to supporting child well‐being, in ways that are emblematic of how relational health is framed in this paper. By building the capacity to provide peer support and improving educator confidence and competence in re‐framing and handling challenging child behavior and intense emotion‐having, this study demonstrates some evidence that HiH approaches could be a sustainable way to contribute to the well‐being of ECE educators, leaders, and related service providers.

### 4.4 Limitations

This study has some limitations. As an early pilot study, the research team used a convenience approach to sampling. This approach resulted in a relatively homogenous sample; the majority of the sample were white women who were highly educated and experienced as educators. While several Black educators initially participated in the course and consented to the study, they did not complete the course. It is worth noting that many of the Black educators involved in the study came from one, publicly funded ECE program. In acknowledging our sample *does not* represent the diversity of ECE settings or the ECE workforce, additional research is needed to determine how educators from minoritized racial and ethnic groups who are also serving children from these same communities that experience multiple vulnerabilities perceive and are impacted by the Foundations Course. Outside of this study, HiH approaches have been implemented nationally and internationally including people from minoritized racial and ethnic groups that work in publicly funded ECE (AMCHP, 2022; Hand in Hand Parenting, 2023). HiH team members who have developed and refined the Foundation Course and HiH tools are similarly from a range of social locations. However, more evaluation is needed to determine the extent to which there is evidence that this course constitutes a culturally and linguistically competent approach that can help fulfill the promise of ECE to contribute to more equitable outcomes for children, families and providers.

The results of the current early pilot study also combine perspectives of those who are new to the Hand and Hand approach entirely with those who have prior exposure to HiH. This introduces a level of bias, potentially skewing the data towards those who are predisposed to appreciate the HiH approach. Future studies will either recruit only educators who are new to HiH or will analyze results from those with prior exposure separately. Nevertheless, this study indicates even educators with prior exposure find value in the Foundations Course. Similarly, the current study does not parse apart the perspectives of individuals based on their role (e.g., educator or director), nor based on their prior training and/or current use of other social‐emotional curricula or approaches, yet these factors may affect their reactions to the HiH content. Importantly, the 2021 feasibility study for the Foundations Course found that educators with a range of experience levels and prior training experiences found the course to be helpful (Coleman et al., 2021).

The Foundation Course does not yet have a measure of fidelity of implementation. Although findings from this study were promising, additional validated, quantitative measures need to be incorporated into future mixed‐methods evaluations of the HiH approach to capture additional constructs of importance. These future directions for research will be undertaken after additional course revision.

## 5 CONCLUSION

The need for building and sustaining a culture of connection has been rising for decades amid mounting awareness of mental health challenges for children and the adults who care for them. In response, the US Surgeon General, Dr. Vivek Murthy urged us all to make “a commitment to invest in and take actions establishing that our connection with others is a core value of this nation” (OSG, 2023, p. 45). The HiH Foundations Course provides the “revolutionary” and destigmatizing framing of emotional expression as functional and necessary for building relationships towards a nested ecology of care. The results of this early pilot study constitute the first steps towards empirically demonstrating the potential of the HiH approach to support early relational health in ECE by building the health, social‐emotional development and learning capacities of adults and children that are vital for improving family, organizational, and community well‐being within a system of care.

## CONFLICT OF INTEREST STATEMENT

The authors have no conflicts of interest to disclose. As a research‐practice partnership, multiple members of the team are affiliated with Hand in Hand Parenting, a nonprofit that administers the Foundations Course. Nevertheless, they do not have financial interests in the outcome of this study, and nor do the other authors.

## Supporting information



Supporting‐Information

Supporting‐Information

## Data Availability

The data that support the findings of this study are available from the first author upon reasonable request.
