# Supplementary material for: Building a culture of connection in early childhood education: The Hand in Hand Foundations Course
Source: Infant Ment Health J. 2025 Jun 23;46(6):675–95. doi: 10.1002/imhj.70030 (PMC12644306; doi:10.1002/imhj.70030)
Supplement: Supplementary file 1 — Supporting‐Information [file IMHJ-46-675-s002.pdf]

Hand in Hand's Foundations Course for Early Childhood Educators

Course Overview and Structure

October 10 - December 12, 2022

Course Description:

The Foundations Course is an 8-week introductory course to the Hand in Hand approach and the listening tools. Over the 8 weeks, you will learn Hand in Hand's theory, all five listening tools, how to use the tools in your specific work setting, and engage in personal reflection. There will be videos and readings for you to complete each week along with a 1-hour weekly mentoring call with 2-5 other early care and education professionals for personalized support and feedback. The mentoring calls will be led by a Certified Hand in Hand Instructor and they will guide you through the 8-week course and lead all of your calls.

Course Structure:

This course is designed as a distance program with weekly zoom support and an online classroom that will open new course materials each week. Every week you will learn about one new tool through a mentoring call and a set of videos and readings for you to complete at your own pace. Please review the materials before attending your mentoring call. However, we encourage you to attend your weekly mentoring calls even if you were not able to review the materials beforehand.

WEEK 1 **Introductions**

(October 10-October 17)

The first mentoring call will focus on getting to know each other, orienting to the course, and reviewing the 8-week agenda.

WEEK 2 **Listening Partnerships**

(October 17-October 24)

This week we'll focus on our own well-being and the ways in which we can all get stretched too far. When we get stretched, often the warmth we want to convey to children can turn artificial, or may be eclipsed by harsh tones. If we are not replenishing our pool of resources and emptying our emotional waste baskets, the joy of children can degrade into formulaic duty or even drudgery. Listening partnerships are a way for us to provide support for each other and replenish our social emotional systems.

WEEK 3 **Staylistening**

(October 24 - 31)

This week we'll explore Staylistening, a powerful tool to support children during emotional moments. This tool is all about listening to a child's feelings with our warm attention and care, while setting limits on off-track behavior.

WEEK 4

Special Time

(October 31 - November 7)

This week we'll explore Special Time. Special Time is one-on-one, child-led playtime. During Special Time the adult follows the child's lead in play. Special Time is labeled by the adult (as Special Time/Kids Rule Time) and is timed. We'll focus on ways in which Special Time can be used in large group settings.

WEEK 5

Setting Limits

(November 7-November 14)

This week we'll explore a new way to set limits. Hand in Hand's Setting Limits is a 3-step process: Listen-Limit-Listen. When a child doesn't feel connected, they can't think. They signal that they can't think with off-track behavior. For good thinking to return they need to feel connected to a well-regulated adult who can set limits on that behavior, listen to the feelings that caused the emotional alarm, and offer opportunities for co-regulation.

WEEK 6

Playlistening

(November 14 - November 21)

This week we'll learn the final tool, a play-based strategy for the classroom called Playlistening. Playlistening is where we take the less powerful role in play and follow the giggles. This can often involve physical play and roughhousing (without tickling). We'll also learn how Playlistening can be used without any physical contact and can be further adapted to adhere to your school's guidelines.

WEEK 7

Connection Plans

(November 28 - December 3)

This week we'll put all the tools together and create personalized Connection Plans for you to implement in your school setting after the course is over.

WEEK 8

Closing & Group Interview

(December 5 - December 12)

The final week we'll bring the course to a close and prepare for optional next steps. Hand in Hand researchers will join for a group interview.

Information on Hand in Hand Parenting:

Hand in Hand Parenting is an international not-for-profit parent support organization founded 33 years ago by Patty Wipfler. Hand in Hand has developed a set of 5 concrete tools that help adults build strong relationships with the children in their lives. The Hand in Hand tools are built on the beliefs that connection is the foundation for emotional and cognitive functioning and that people recover from difficult experiences in the context of relationships that allow for play, emotional expression, and co-regulation.

The 5 concrete Hand in Hand tools are called "Listening Tools" because they help adults listen to children in the context of play, high emotions, and setting limits; and they help adults listen to each other to build good support for their own caregiving.

*Hand in Hand is now designated as a "Promising Practice" by the Association of Maternal and Child Health Programs (AMCHP), a national registry of evidence-based programs in the US. A Promising Practice within the public health space exponentially expands Hand in Hand's reach and service to family services, federal preschool programs, center-based programs, as well as higher education programs across the country that train and serve families, educators, and allied professionals. This is the first national recognition to come out of Hand in Hand's Research and Development (R&D) arm. The new R&D team engages in rigorous scientific studies documenting the impact of the Hand in Hand approach for families and professionals across the globe.

https://www.handinhandparenting.org/research
