# Supplementary material for: Building a culture of connection in early childhood education: The Hand in Hand Foundations Course
Source: Infant Ment Health J. 2025 Jun 23;46(6):675–95. doi: 10.1002/imhj.70030 (PMC12644306; doi:10.1002/imhj.70030)
Supplement: Supplementary file 2 — Supporting‐Information [file IMHJ-46-675-s001.docx]

**Appendix B.** Summary of Qualitative Findings Pertaining to the Benefits and Challenges of the Foundations Course

| **Benefits** | **Challenges and Barriers** |
| --- | --- |
| ***Course overall:*** | |
| - Educators feel more confident and less stressed interacting with children demonstrating strong emotions - Educators have a new understanding of children’s emotions and their role when they are expressed - Educators feel relieved - Educators can respond to children’s emotions in a calmer manner - Educators form more meaningful connections with children and families - Teaching teams collaborate better - Educators report improved self-awareness - Educators may share the tools with families | - Too short to produce deep enough understanding of the tools for all participants to implement them as intended - Difficult to implement if colleagues are not also trained in HiH and bought in to the approach - Revised and tailored course materials needed. Examples include step-by-step guides for each tool and examples of each tool used in ECE contexts with children of different ages and needs |
| ***Specific tools:*** | |
| **Listening Partnerships:**   - Feeling supported, safe, unburdened, and better able to listen to others | **Listening Partnerships:**   - Difficulty identifying the right person, having enough time, knowing how to initiate a partnership, and feeling awkward |
| **Staylistening:**   - Improved relationships with children, more insight into children, reduced off-track behavior | **Staylistening:**   - Lack of time and other staff to attend to other children |
| **Setting Limits:**   - Easy to learn, effective, provides sense of relief for educators, helps educators stay calm | **Setting Limits:**   - None described; because Setting Limits is part of Staylistening, the same challenges should apply |
| **Special Time:**   - Improved closeness with children, fun, a break from being in charge | **Special Time**:   - Can seem too time-consuming, may not have enough staff to allow for extended 1:1 attention, may be challenging to follow children’s lead, other children may want attention at the same time |
| **Playlistening:**   - Helps ease tensions with children, encourages on-track behavior, fun | **Playlistening:**   - May feel awkward |
